# A droplet digital PCR assay targeting 16 human papillomavirus genotypes

**DOI:** 10.1099/acmi.0.001166.v3

**Published:** 2026-09-02

**Authors:** Gerald L. Murray, Prisha Balgovind, Sepehr N. Tabrizi, Brian J. Morris, Peter French, Emel Yilmaz, Laila Sara Arroyo Mühr, Suzanne M. Garland

**Affiliations:** 1 Department of Obstetrics, Gynaecology and Newborn Health, University of Melbourne, Melbourne, Vic, Australia; 2 Centre for Women’s Infectious Diseases, The Royal Women’s Hospital, Melbourne, Vic, Australia; 3 Murdoch Children’s Research Institute, Melbourne, Vic, Australia; 4 Department of Obstetrics and Gynaecology, University of Western Australia, Perth, Australia; 5 School of Medical Sciences, Faculty of Medicine and Health, The University of Sydney, Sydney, NSW, Australia; 6 Innlanz Limited, Sydney, Australia; 7 International HPV Reference Center, Center for Cervical Cancer Elimination, Karolinska Institutet and Karolinska University Hospital, F56, 141 86 Stockholm, Sweden

**Keywords:** cervical cancer, human papillomavirus, PCR, viral load

## Abstract

**Background.** Cervical screening with high-precision assays such as human papillomavirus (HPV) DNA testing is essential for the detection and treatment of precancerous lesions. HPV genotypes have different oncogenic potential and require different clinical management, illustrating the importance of extended genotyping. HPV quantification has demonstrated clinical relevance in both diagnosis and treatment.

**Objective.** To develop a droplet digital PCR assay for the detection and quantification of 16 HPV genotypes, with comparison to a commercial test and validation on clinical samples.

**Methods.** Primers and probes were designed to target the E6 region of 16 HPV genotypes: 16, 18, 31, 33, 35, 39, 45, 51, 52, 56, 58, 59, 66, 68, 73 and 82. Each target was evaluated to assess performance and reliability using synthetic DNA constructs, quantified international reference standards and clinical screening samples (*n*=303) genotyped using the Seegene Anyplex II HR HPV Detection assay.

**Results.** Each assay demonstrated high target specificity, without cross-reactivity observed among the HPV genotypes selected. Using international molecular standards, the assay reliably detected high-risk genotypes across serial dilutions, with detection down to the level of one international unit or genome equivalent per microlitre. When applied to clinical samples with and without a histological diagnosis of cervical intraepithelial neoplasia 2 or worse (CIN2+), the assay reliably detected key HPV genotypes, with the exception of 7 of 234 (3%) of HPV-positive samples, all of which exhibited very low viral load.

**Conclusion.** Although previous studies have described digital PCR methods targeting HPV E6, they have typically focused on a limited number of genotypes. This study expands upon existing methodology by introducing a sensitive and specific method for detection and quantification of 16 high-risk and potentially high-risk HPV genotypes.

## Data Summary

All data relating to this study are presented in the manuscript and supplementary file.

## Introduction

Human papillomavirus (HPV) infection is the underlying cause of multiple cancers of the anogenital region and oropharynx [1, 2]. Among the more than 200 HPV genotypes identified, 12 HPV genotypes (HPV16, 18, 31, 33, 35, 39, 45, 51, 52, 56, 58 and 59) have been classified as Group 1 carcinogens by the International Agency for Research on Cancer, with HPV68 designated as probably carcinogenic (Group 2A) [3]. HPV66 was previously listed as probably carcinogenic but was subsequently downgraded to possibly carcinogenic (Group 2B) [4]. Additionally, a recent global systematic analysis of over 111,000 cases further identified genotypes 26, 69, 73 and 82 as having a small but statistically significant contribution to cervical cancer cases [5].

The HPV double-stranded DNA genome encodes two capsid proteins (L1 and L2) and six early genes (E1, E2, E4, E5, E6 and E7) [2]. Early PCR was developed targeting HPV E6 of HPV genotypes 16, 18, 33, 6 and 11 [6, 7]. While many commercial diagnostic tests target the L1 region of HPV, deletions in this region have been reported in a number of HPV genotypes, especially after integration into the host genome [8–10], emphasizing the importance of a breadth of assays with alternative detection targets.

Digital PCR offers a robust platform for quantifying DNA targets and has reduced susceptibility to PCR inhibitors commonly found in biological samples [11]. Several studies have used droplet digital PCR (ddPCR) for HPV detection in HPV-associated diseases [12–20]. However, these assays have generally been limited in genotype coverage, with most studies targeting only a few genotypes. Examples include L1-gene-based assays for HPV genotypes 11, 16, 18 and 45 [15]; the E6-gene-based assays for 16, 18, 33 and 45 [14]; 31, 35, 39, 51 and 56 [16]; and E7-gene-based assays for HPV genotypes 16 and 18 [13].

Viral load is of research interest and has clinical relevance. For example, circulating HPV DNA levels in blood can be indicative of tumour stage and size [13] and can assist with monitoring response to treatment [18, 19]. In HPV-related anal cancer, viral load has been associated with lesion grade [21] and patient survival [20, 22, 23]. In cervical cancer, the impact of load is subject to some controversy and requires further investigation [24], for example, load is not yet confirmed as a biomarker for progression, partly owing to the lack of quantification studies and assays to quantify key HPV types. In the current study, we present ddPCR detection assays targeting the E6 gene across a broad panel of high-risk and potentially high-risk HPV genotypes.

## Methods

### DNA template

Synthetic DNA constructs of ∼1 kb encompassing the E6 and E7 HPV genomic regions were designed for each HPV genotype from the reference sequences listed in Table 1 (gBlocks; Integrated DNA Technologies, Singapore). International reference standards were obtained as a 10-fold dilution series from the International HPV Reference Center (Stockholm, Sweden). Clinical samples (*n*=303) were obtained from the routine screening programme in Sweden, comprising 100 cases with a histological diagnosis of cervical intraepithelial neoplasia 2 or worse (CIN2+) and 203 controls.

**Table 1.** Primer and probe sequences^*^

Genotype	PaVE reference^†^	Name^‡^	Sequence	Product size (bp)
16	333031	B_P16	/5HEX/CGCAGTAAC/ZEN/TGTTGCTTGCAGTACACA/3IABkFQ/	102
		B_16F	GCACAGAGCTGCAAACAACT	
		B_16R	AATCCCGAAAAGCAAAGTCA	
18	60975	C_P18	/5HEX/CCTCTGTAA/ZEN/GTTCCAATACTGTCTTGCAA/3IABkFQ/	143
		C_18F	TGTGCACGGAACTGAACACT	
		C_18R	TGCAGCATGGGGTATACTGT	
31	333048	A_P31	/5HEX/CCAATGCCG/ZEN/AGCTTAGTTCATGCA/3IABkFQ/	104
		A_31F	TGCAGAAAGACCTCGGAAAT	
		A_31R	TTTCTGTTAACTGACCTTTGCAG	
33	333049	B_P33	/5HEX/TGCATTCCA/ZEN/CGCACTGTAGTTCAATG/3IABkFQ/	144
		B_33F	TCAAGACACTGAGGAAAAACCA	
		B_33R	AAATCTGCAAATGCAAAATCA	
35	396997	A_P35	/5HEX/CCGACCTGT/ZEN/CCACCGTCCAC/3IABkFQ/	185
		A_35F	AGTGTGTATGGAGAAACGTTAGAAA	
		A_35R	GTTGGTTTCCAACAGGACATAC	
39	333245	C_P39	/5HEX/TGTGCACAA/ZEN/CGCTGGACACCA/3IABkFQ/	91
		C_39F	GCGCGATTTCACAATCCT	
		C_39R	AGGCTATTGTAATGTCCTGCAA	
45	397022	C_P45	/5HEX/CTGGCACCG/ZEN/CAGGCACCTTA/3IABkFQ/	129
		45F	AAACTCTGTATATGGAGAGACACTGG	
		45R	CGTTTGTCCTTAAGGTGTCTAC	
51	333087	D_P51	/5HEX/CGTCCCGCT/ZEN/ATTTCATGGAACC/3IABkFQ/	103
		D_51F	GCAAAAATTGGTGGACGAAA	
		D_51R	GGTTTCGTTACGTTGTCGTG	
52	397038	A_P52	/5HEX/TGCACTGCA/ZEN/CACACTGCAGCC/3IABkFQ/	160
		A_52F	TGAGGTGCTGGAAGAATCG	
		A_52R	AAAGCGTAGGCACATAATACACAC	
56	397053	D_P56	/5HEX/CGAAGCCT/ZEN/GCACCACTTGAGTGA/3IABkFQ/	189
		D_56F	TTCAACAATCCACAGGAACG	
		D_56R	TCTGCACACTGCATAAGGAAA	
58	222386	B_P58	/5HEX/CGCTATATG/ZEN/GAGACACATTAGAACAAACAC/3IABkFQ/	205
		B_58F	TGTAAAGTGTGCTTACGATTGC	
		B_58R	GACCCGAAATATTATGAAACC	
59	557236	E_P59	/5HEX/TGCATTTCA/ZEN/GACACGCTGCATACG/3IABkFQ/	159
		E_59F	GGGGAACTGCAAGAAAGAGA	
		E_59R	TGTTTCTCCATACACGGAATCT	
66	1020290	P66	/5HEX/AGCCTGCAC/ZEN/CATCTGAGCGA/3IABkFQ/	189
		66F	TCAGCAATACACAGGAACGTC	
		66R	CCCTACATACTGCATATGGCC	
68	71726685	D_P68	/5HEX/TGCAGAAGG/ZEN/CAACTACAACGGACA/3IABkFQ/	150
		D_68F	CTGTGCAGGACATTGGACAC	
		D_68R	TTGGCATGCAGCAAATGGTA	
73	1491692	E_P73	/5HEX/CTTCGTCAC/ZEN/ATAACGCTTGTAGCTTG/3IABkFQ/	97
		E_73F	CCAATTCAGAAGAACGACCA	
		E_73R	CGTTGGCAAAACACACAGTC	
82	6970427	E_P82	/5HEX/TCAGGCCCA/ZEN/AGTGGTCTCTGACA/3IABkFQ/	193
		E_82F	CCATATGCAGCATGCAAAAA	
		E_82R	TTTTGTCGTCCACCACCTTT	

*Primers and probes were supplied by Integrated DNA Technologies (Singapore). Estimated primer Tm 58–61 °C, estimated probe Tm 68–70 °C.

†Reference genome from the papillomavirus episteme (PaVE) database.

‡The prefix letter in the primer name indicates potential compatibility for multiplexing (not evaluated in this study).

5HEX, 5′ Hexachlorofluorescein; 3IABkFQ, 3′ Iowa Black® FQ.

Samples were collected using a cervical brush and suspended in ThinPrep medium (Hologic). DNA was extracted from 200 µl of sample using the Roche MagNA Pure 96 DNA and Viral NA Small Volume Kit (Roche Diagnostics GmbH, Germany) and then eluted in 100 µl of elution buffer. Five microlitres of extract was tested using Anyplex™ II HPV HR Detection (Seegene, South Korea), which detects 14 HPV types (high-risk and potential high-risk genotypes 16, 18, 31, 33, 35, 39, 45, 51, 52, 56, 58, 59, 66 and 68) with a semiquantitative indication of signal strength for positive samples from ‘+’ to ‘+++’. This assay is based on tagging oligonucleotide cleavage and extension (TOCE) technology and has a previously reported detection limit of 50 copies per PCR [25].

### Digital PCR assay

Primers and probes used in this study were supplied by Integrated DNA Technologies (Singapore) (Table 1). PCR was performed using the BioRad droplet digital PCR system according to the manufacturer’s protocols, with ddPCR™ Supermix for Probes (No dUTP), 500 nM primers and 250 nM probe, and 5 µl of DNA template in a 20 µl volume. Droplets were generated with a BioRad QX200™ Droplet Generator. PCR conditions included an activation step at 95 °C for 10 min and then 45 cycles of three steps (94 °C for 30 s, 55 °C for 10 s and 60 °C for 60 s), followed by a final step of 98 °C for 10 min. Reactions were analysed on the BioRad QX100™ ddPCR system. International standards (HPV genotypes 16, 18, 31, 33, 35, 39, 45, 51, 52, 56, 58 and 59) were analysed in single or duplicate and averaged, while clinical samples were analysed once (HPV genotypes 16, 18, 31, 33, 35, 39, 45, 51, 52, 56, 58, 59, 66 and 68). A noted limitation of digital PCR is the reduced dynamic range compared to quantitative PCR, and some samples required dilution prior to analysis. Digital PCR provides a direct measurement of DNA copies reported as copies per reaction. For this study, results were not normalized to a human reference gene.

A coefficient of variance analysis was performed by analysing assays for each target (minimum of triplicate), dividing the standard deviation by the mean (expressed as a percentage). The limit of blank was determined as described by BioRad (https://www.bio-rad.com/en-au/resources/statistical-tools-limit-blank-limit-detection) using the results of 45 blank assays.

## Results and discussion

### Digital PCR design

**Fig. 1.**
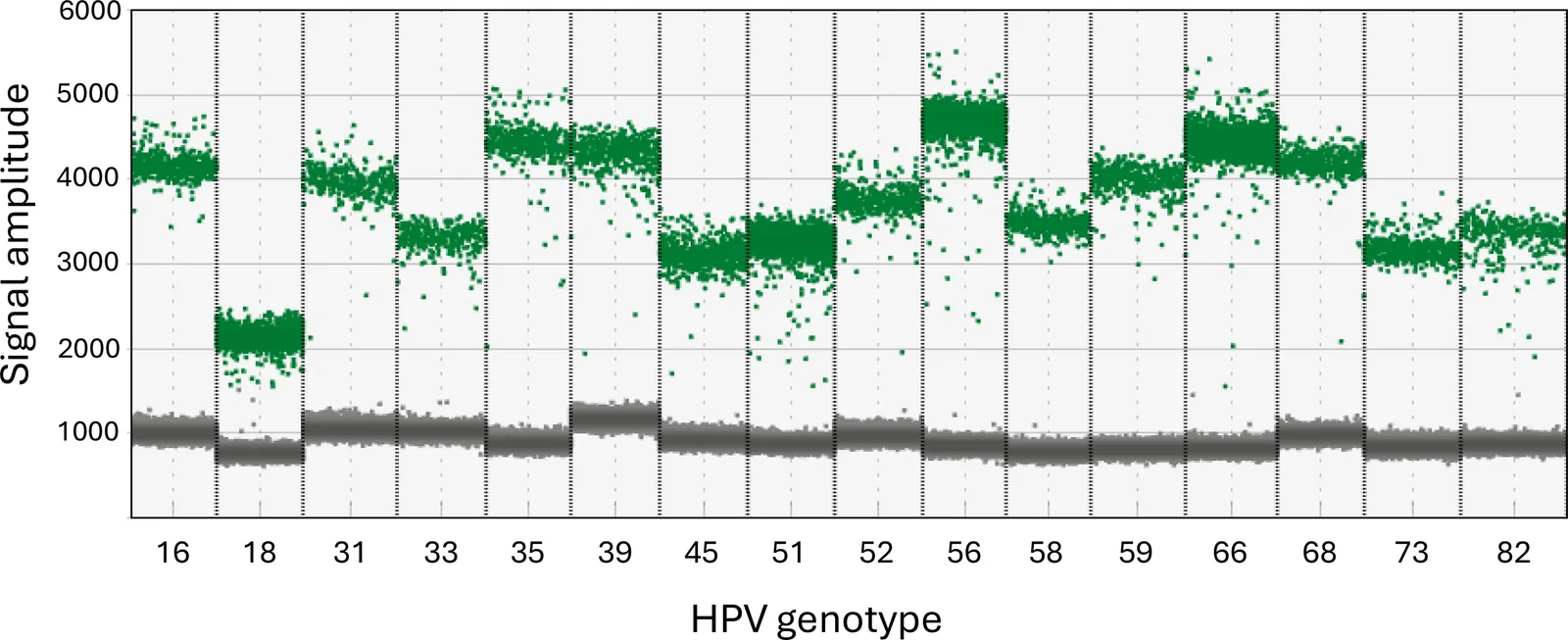
Representative ddPCR assay analysis for 16 HPV types. Synthetic DNA templates were used for this example.

PCR primers targeting the 5′ region of the E6 gene were designed for 16 HPV genotypes associated with anogenital cancers (Table 1), each paired with a genotype-specific fluorescent hydrolysis probe. Amplicon sizes ranged from 91 to 205 bp. When tested with synthetic DNA constructs, each ddPCR assay detected the corresponding genotype with clear signal separation between positive and baseline-negative droplets (Fig. 1). Assay reproducibility was determined through coefficient of variance for the 12 most important types (Table 2), with an average of 5.4 (standard deviation of 3.8). A limit of blank calculation was performed and determined to be 0.07 copies per microlitre.

**Table 2.** Analysis of international standards for 12 high-risk HPV genotypes by ddPCR, and determination of coefficient of variance

	Concentration determined by ddPCR (copies/μl)											
Standard concentration*	HPV16	HPV18	HPV31	HPV33	HPV35	HPV39	HPV45	HPV51	HPV52	HPV56	HPV58	HPV59
1	1.075	0.657	1.186	1.614	0.65	0.66	1.019	1.944	1.463	2.700	2.225	1.73
10	12.10	8.96	3.32	14.32	11.40	9.16	11.82	22.60	25.00	18.46	24.20	9.14
100	132.2	74.4	30.8	129.8	112.2	96.2	99.2	218.6	215.6	208.8	250.2	131.8
1,000	1,298	680	349	1,459	1,029	950	1,018	2,250	2,357	2,157	2,380	1,230
10,000	13,041	7,153	3,315	14,886	10,391	9,406	10,746	22,600	22,440	22,315	21,343	12,818
100,000	132,891	65,466	30,283	132,652	105,590	90,403	102,738	na†	231,063	na ^b^	225,959	124,844
Coefficient of variance‡	6.87	2.37	3.43	3.43	6.51	1.75	8.13	5.36	5.27	15.7	3.74	2.37

*Standards were provided as international units/μl (HPV 16, 18, 31, 33, 45, 52 and 58) or genome equivalents/μl (HPV 35, 39, 51, 56 and 59). Five microlitres of standard was used, except for the highest concentration sample (typically 1 µl used). Pearson correlation for detection of each HPV genotype was >0.999.

†Results were not available for HPV51 and HPV56 at the highest concentration standard due to saturation of the detection system.

‡Coefficient of variance was determined in a separate set of assays (in a minimum of triplicate) using reference standards.

To assess cross-reactivity, each primer/probe combination was analysed for cross-reactivity against the other 15 genotypes. Sixteen DNA pools were assembled, each containing 15 genotypes, and subjected to ddPCR using the assay specific to the genotype omitted. No amplification was detected for the other genotypes, confirming high specificity and absence of cross-reactivity.

To evaluate performance in the context of multiple HPV genotypes, a DNA pool containing all 16 E6 DNA constructs was used as a template. Each assay successfully detected its corresponding genotype within the mixed pool.

### Analysis of standards and determination of detection limit

Analytical validation was conducted using available international standards for 12 high-risk HPV genotypes. Standards were supplied in a 10-fold dilution series corresponding to a range from 100,000 down to 1 international unit per microlitre or genome equivalent per microlitre. The ddPCR assay demonstrated high accuracy across the full concentration range, with a high linear correlation between expected and observed values (Table 2).

### Analysis of clinical samples for detection of genotypes

The performance of the digital PCR assay for genotype detection in clinical samples was evaluated using clinical specimens genotyped with an established diagnostic assay, the Anyplex II HR HPV Detection. Those samples, collected as part of a separate study, encompassed a broad range of viral concentrations. From 303 samples, 234 tested positive for genotypes of interest by the reference assay, and 14 genotypes were subsequently analysed using ddPCR (Table 3). A comparison of reference assay signal strength and viral DNA concentration is available in the online Supplementary Material Fig. S1. Overall, ddPCR demonstrated good concordance with the reference assay, with >94% agreement for 11 of the 14 genotype targets (overall agreement of 227/234, 97%). Instances of non-detection by ddPCR corresponded to samples with very low signal intensity in the reference assay (reported as ‘+’), suggesting a low viral load. The lowest concordance was observed for HPV66, but notably more than half of the HPV66-positive samples had very low viral loads (Table 2). These discrepancies likely reflect stochastic sampling effects near the assay’s limit of detection, resulting in a false-negative result. Given the limited clinical relevance of HPV66, reduced sensitivity near the limit of detection is unlikely to affect the intended research or clinical applications of the assay.

**Table 3.** Comparison of genotype detection by digital PCR and the reference assay

Genotype	Samples positive by ddPCR/reference (%)*	ddPCR detection of samples with very low load†
HPV16	45/45 (100)	7/7
HPV18	10/10 (100)	1/1
HPV31	26/27 (96.3)	3/4
HPV33	14/14 (100)	3/3
HPV35	11/11 (100)	1/1
HPV39	17/18 (94.4)	5/6
HPV45	10/11 (90.9)	3/4
HPV51	11/12 (91.7)	4/5
HPV52	21/21 (100)	8/8
HPV56	15/15 (100)	3/3
HPV58	20/20 (100)	4/4
HPV59	6/6 (100)	1/1
HPV66	6/9 (66.7)	2/5
HPV68	15/15 (100)	5/5
Total	227/234 (97.0)	50/57 (87.7)

*Reference assay: Anyplex II HR HPV Detection (Seegene). Note that the missed detections for HPV31, HPV39, HPV45, HPV51 and one of HPV66 occurred in cases with mixed infection; in each sample a second HPV genotype (either HPV16 or HPV52) was detected at very high levels and is most likely responsible for the lesion.

†Samples defined by the minimal reading of ‘+’ in the reference assay.

Study limitations include the lack of quantitated standards for four of the genotypes analysed. Due to the large number of genotypes covered by the PCR assay, there were limited numbers of clinical samples that were positive for each individual genotype. This method is best applied not as a screening assay but as a targeted approach in samples with known HPV genotypes present (e.g. as a reflex test on a genotyped sample or for analysing a series of samples from a patient). The use of multiplexing (not explored in this study) would reduce labour and reagents, with some digital systems permitting up to five channels (i.e. targets) per instrument.

## Conclusion

This study developed an assay for reliable detection of 16 HPV genotypes without cross-reaction, including the 12 ‘preferred’ genotype targets listed in the WHO HPV screening Target Product Profile [26] plus an additional 4 genotypes. This assay differs from other digital PCR methods in both the gene target and the extensive panel of genotypes, making it useful for research requiring accurate quantification of viral load and extended genotyping. It may also be useful in future clinical approaches to help guide treatment decisions.

## Supplementary material

10.1099/acmi.0.001166.v3 Fig. S1.

## Abbreviations

CIN2+: cervical intraepithelial neoplasia 2 or worse
ddPCR: droplet digital PCR
HPV: human papillomavirus
PaVE: papillomavirus episteme
TOCE: tagging oligonucleotide cleavage and extension
