# Supplementary material for: A droplet digital PCR assay targeting 16 human papillomavirus genotypes
Source: Access Microbiol. 2026 Sep 2;8(9):001166.v3. doi: 10.1099/acmi.0.001166.v3 (PMC13537474; doi:10.1099/acmi.0.001166.v3)
Supplement: Supplementary file 1 [file acmi-8-01166-s001.pdf]

Supplementary figure. HPV loads for each genotype determined by digital PCR plotted against Seegene Anyplex II HR HPV Detection assay signal. The Seegene assay signal has three output levels of 1+, 2+, or 3+. HPV concentration is expressed in copies per 5 µL of DNA extract, equivalent to the amount of material used in the Seegene PCR assay. To permit plotting on a logarithmic scale, any digital PCR values of zero were converted to 1.

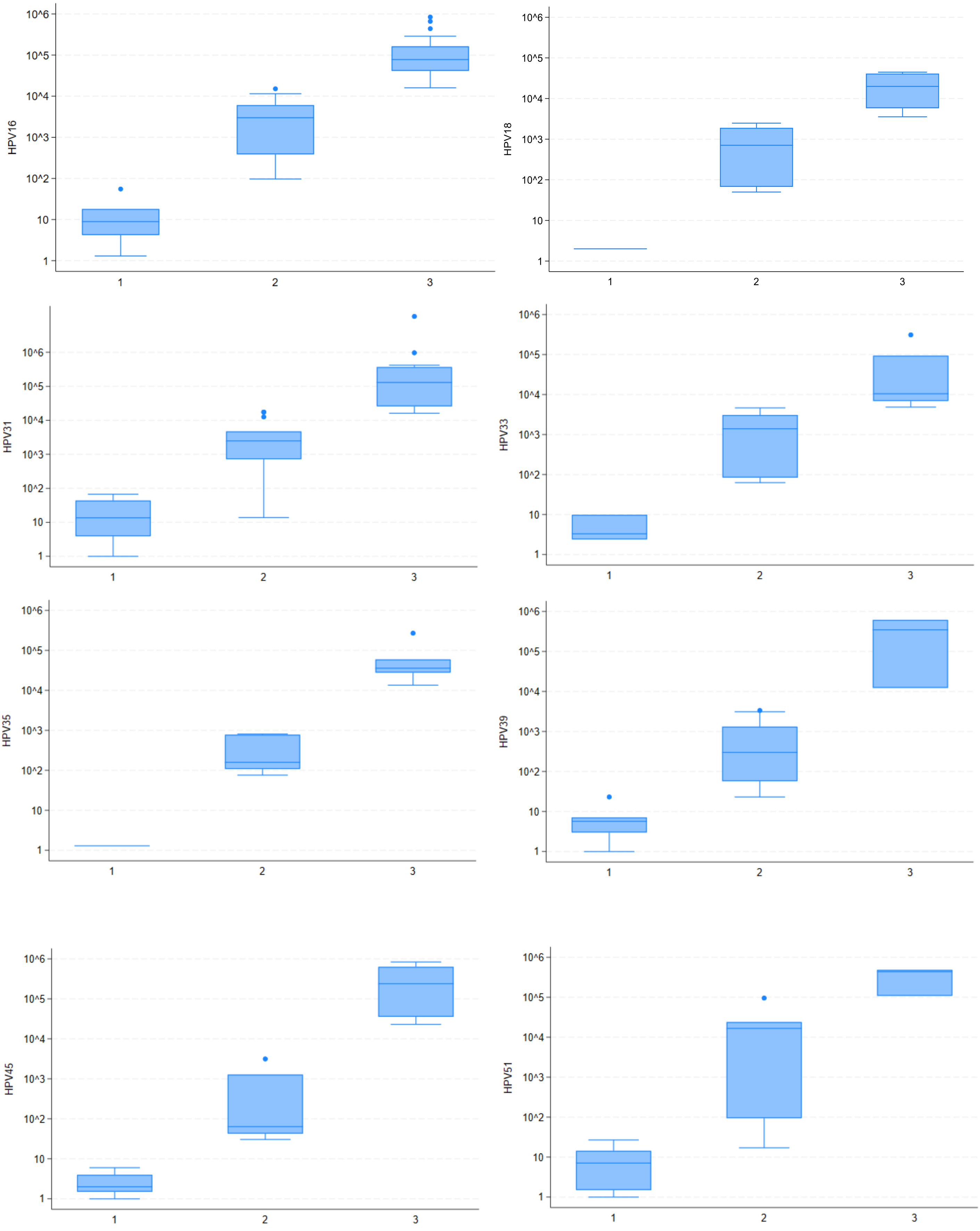

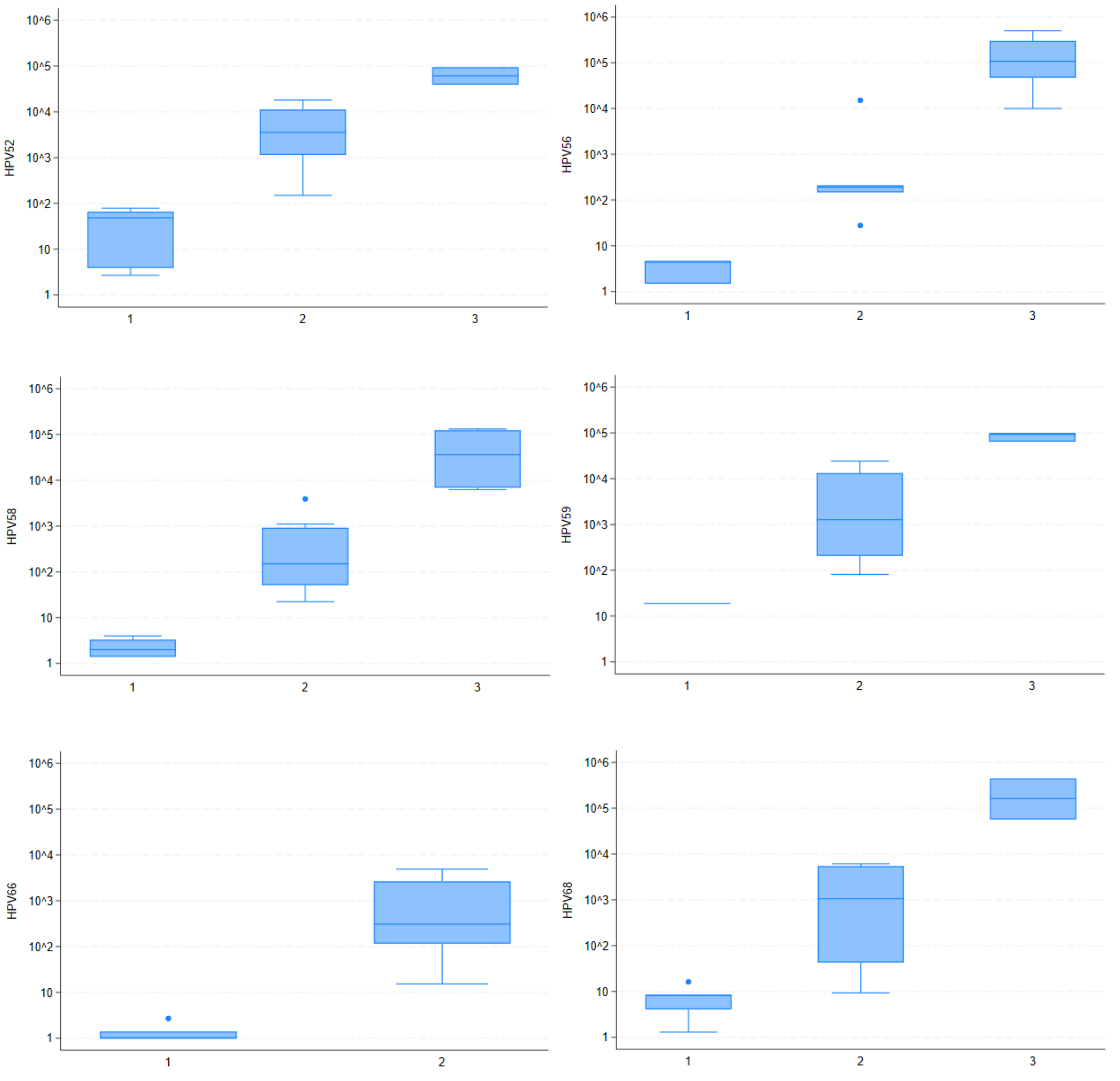
HPV52
HPV56
HPV58
HPV59
HPV66
HPV68
10^6
10^5
10^4
10^3
10^2
10
1
1
2
3
